# Additional primary malignancies in a Polish cohort of uveal melanoma patients: a review of 644 patients with long-term follow-up

**DOI:** 10.1186/s12886-023-03246-z

**Published:** 2023-12-12

**Authors:** Marta Wróblewska-Zierhoffer, Barbara Paprzycka, Anna Kubiak, Łukasz Tomczyk, Iwona Rospond-Kubiak

**Affiliations:** 1 Department of Ophthalmology, Poznań University of Medical Sciences, Poznań, Poland; 2 Polyclinic of Plastic and Ophthalmic Surgery, Kobylniki, Poland; 3 Department of Ophthalmology, Poznań District Hospital, Poznań, Poland; 4 Greater Poland Cancer Registry, Greater Poland Cancer Centre, Poznań, Poland; 5 Department of Food Safety and Quality Management, Poznań University of Life Sciences, Poznań, Poland

**Keywords:** Eye, Malignant Neoplasm, Uveal Melanoma, Second primary carcinoma

## Abstract

**Aim:**

To investigate the frequency and location of additional primary malignancies in a Polish cohort of uveal melanoma (UM) patients registered in a single centre database.

**Material and method:**

Retrospective data analysis of patients treated for uveal melanoma at the Department of Ophthalmology, Poznań University of Medical Sciences, Poland between 1991 and 2017. Data on the diagnosis of the additional malignancies were obtained during the follow-up visits in our Department and/or from the Greater Poland Cancer Registry. The exclusion criteria comprised no confirmed follow-up or incomplete clinical entry data.

**Results:**

Among 644 UM patients registered in the database up to 2017, the additional malignancy was diagnosed in 126 (20%) patients: 71 men, 55 women at the median age of 67 years (range: 34–94). In 48 patients (38%), the additional malignancy occurred prior to the diagnosis of UM, in 73 (58%) patients - after it. The most common locations of second cancer were skin (20 cases / 15%), breast (17 cases / 13%) and lungs (15 cases / 12%). The median follow-up was 36 months (range: 3–242). 87 patients (69%) died by the study close, 32 (37%) of them due to metastatic disease from uveal melanoma, 41 (47%) due to another cancer.

**Conclusions:**

The frequency of additional primary malignancies was higher in our cohort than reported by most of other groups. If there is a certain predisposition to a specific type of additional primary carcinoma in UM patients, the analysis of larger database is required.

## Introduction

Uveal melanoma (UM) is the most common primary intraocular malignancy in adults with the incidence of about 6–7 cases per 1 mln white Caucasians, with a similar frequency in both sexes. There are, however, some reports that the incidence of UM might be rising specifically in north of Europe, even up to 8 cases per million in Ireland, Norway and Denmark [1–4]. Uveal melanoma (UM) occurs most often in older patients, usually in the 6-7th decade of life. Choroid is affected in more than 90% cases, ciliary body in 6% and iris in 4%, the tumour is usually unilateral. [3, 5]. Risk factors associated with the UM development include fair skin colour, light-coloured irises (blue or grey), tendency to sunburn, congenital atypical mole syndrome, ocular melanocytosis (nevus of Ota), or BAP1/MBD4-tumour predisposition syndrome [5–10].

Most patients present with symptoms like blurred vision, photopsias, metamorphopsias, scotomas or painful eye. However, up to 30% of UMs can be found accidentally during routine examination due to the asymptomatic course [1, 3]. Currently the gold-standard for treatment is radiotherapy [2, 11–13]. However, despite prompt treatment, even up to 50% of UM patients would develop metastatic disease within 5 years from the diagnosis, with the liver being most common site of metastasis [6, 14].

Additional primary malignancies is a term referring to other neoplasms that may be related to already existing cancers (both treated and untreated), but develop independently, not as a recurrence or metastasis of primary disease [13, 15]. It is of note that, according to some previous reports, the additional primary neoplasms may occur more frequently within uveal melanoma patients [10, 13, 16–19], with the breast cancer being the most common location [20].

## Aim

The purpose of this study was to look at the frequency of additional primary malignancies among UM patients treated throughout the period of over 20 years in a single institution.

## Materials and methods

The medical records of the patients treated for the uveal melanoma at the Department of Ophthalmology, Poznań University of Medical Sciences, between 1991 and 2017, were retrospectively reviewed. The exclusion criteria comprised undocumented follow-up and incomplete clinical entry data. The data regarding the diagnosis of other malignancy, patients’ survival and cause of death were obtained during the follow-up visits at our Department and/or from Greater Poland Cancer Registry, operating at Greater Poland Cancer Centre, Poznań, where the majority of our patients received their oncologic treatment.

The study adhered to the tenets of the declaration of Helsinki and was approved as a part of retrospective study by Bioethics Committee at Poznań University of Medical Sciences.

The statistical analysis was performed with Statistica 13.1 software. The Shapiro–Wilk test was used to check for normality of distribution. The student’s t-test was used to compare quantitative variables. Pearson’s chi-squared test was used to check the relationship between qualitative variables. The level of statistical significance was set at *p* < 0.05.

## Results

A total of 658 UM patients were registered in the database, as they received their primary treatment at our institution between 1991 and 2017. 14 patients were excluded due to uncomplete follow-up data and ultimately, 644 UM patients were included in the analysis: 334 women (52%) and 310 men (48%), at the median age of 62 years (range:16 to 98).

Out of the 644 cases, 126 (20%) patients have been diagnosed with additional primary malignancy: 55 women (44%) and 71 men (56%). The median age in this cohort of patients was 67 years (range: 34 to 94 years), their demographic data are listed in Table 1. The treated melanomas were stage T1 in 19 cases (15%), 43 (34%) were stage T2, 46 (37%) were stage T3 and 18 (14%) were stage T4. The mean largest basal diameter (LBD) of the tumour was 12,24 mm (2,66 − 20,0 mm), with a mean thickness of 7,75 mm (1,23 − 18,10 mm).

**Table 1:** Demographic characteristic of 126 μm patients with additional primary malignancies

Gender, n (%)	
Women	55 (44%)
Men	71 (56%)
**Age, years**	
Mean	66,3
Median (range)	67 (34–94)
**Largest basal diameter (mm)**	
Mean	12,24
Median (range)	12,12 (2,66 − 20,0)
**Tumour height (mm)**	
Mean	7,75
Median (range)	6,73 (1,23 − 18,1)
**TNM categories, n (%)**	
T1	19 (15%)
T2	43 (34%)
T3	46 (37%)
T4	18 (14%)

The median time from the UM treatment to the end of follow-up was 36 months (range: 3 to 242 months). During the period of observation, 87 patients (69%) died, 41 (47%) due to other carcinoma, 32 (37%) due to metastatic spread of uveal melanoma, in 14 cases (16%) the cause of death was not related.

The most common location of the additional primary malignancy in the entire group was skin (20/126, 15%), followed by breast (17/126, 13%) and lungs (15/126, 12%), and with regard to gender – breast in women (16/55, 29%) and prostate in men (14/71, 20%). The percentages and all other locations of additional carcinomas are listed in Table 2. 13 patients (2%) have reported the appearance of third location of primary cancer, however, due to a small size of the group, this subset was not investigated further.

**Table 2:** The location of additional malignancies in 644 μm patients from the studied cohort

ICD-10 code	Location	Total		Women		Men	
		n	%	n	%	n	%
C00	**Lip**	2	2%	1	2%	1	1%
C16	**Stomach**	5	4%	1	2%	4	6%
C18	**Colon**	10	8%	5	9%	5	7%
C20	**Rectum**	3	2%	1	2%	2	3%
C25	**Pancreas**	1	1%	0	0%	1	1%
C32	**Larynx**	3	2%	1	2%	2	3%
C34	**Bronchus and lung**	**15**	**12%**	5	9%	**10**	**14%**
C43	**Malignant melanoma of skin**	**3**	**2%**	**2**	**4%**	**1**	**1%**
C44	**Other skin malignancies**	**17**	**13%**	**6**	**11%**	**11**	**15%**
C45	**Mesothelioma**	1	1%	1	2%	0	0%
C49	**Malignant neoplasm of other ** **connective and soft tissue**	1	1%	1	2%	0	0%
C50	**Breast**	**17**	**13%**	**16**	**29%**	1	1%
C51	**Vulva**	1	1%	1	2%	0	0%
C53	**Cervix uteri**	1	1%	1	2%	0	0%
C54	**Corpus uteri**	6	5%	**6**	**11%**	0	0%
C56	**Ovary**	3	2%	3	5%	0	0%
C61	**Prostate**	14	11%	0	0%	**14**	**20%**
C64	**Kidney**	3	2%	0	0%	3	4%
C67	**Bladder**	10	8%	0	0%	**10**	**14%**
C73	**Thyroid gland**	2	2%	2	4%	0	0%
C83	**Non-follicular lymphoma**	1	1%	1	2%	0	0%
C85	**Other specified and unspeified types of non-Hodgkin lymphoma**	1	1%	0	0%	1	1%
C91	**Lymphoid leukemia**	1	1%	0	0%	1	1%
C92	**Myeloid leukemia**	2	2%	0	0%	2	3%
D03	**Melanoma in situ**	1	1%	0	0%	1	1%
D09	**Carcinoma in situ of bladder**	1	1%	1	2%	1	1%
D45	**Polycythemia vera**	1	1%	0	0%	1	1%
		**126**		**55**		**71**	

While analysing the group in terms of the occurrence of additional primary malignancy, we found out that it was related to gender (p = 0,039) and age at the primary treatment (p = 0,00004). Other factors (largest basal diameter, thickness of the tumour, TNM, stage or histological type of uveal melanoma, co-existence of arterial hypertension and diabetes mellitus ) turned out to be of no significance. Additional statistics are presented in Table 3.

**Table 3:** Subgroups analysis – depending on occurrence of additional malignancies

	Total			With additional malignancies	Without additional malignancies	***p-value***
	N = 644			N = 126	N = 518	
**Gender**						
**Women**	**334 (51%)**			55 / 44%	279 / 52%	**0,03967** ^*^
**Men**	**310 (48%)**			71 / 56%	239 / 48%	
**Age (years)**						
**Median**	62			67	61	0,00004**
**Mean (± SD)**	61			66,29 (± 10,95)	59,77 (± 12,71)	
**Tumours largest basal diameter (mm)**						
**Mean (± SD)**	12,24			12,24 ± 3,72	12,23 ± 3,21	0,98401**
**Tumours height (mm)**						
**Mean (± SD)**	7,57			7,74 ± 2,41	7,52 ± 3,21	0,5706**
**Histological type**						
**Epithelial cell**	66 /16%			15 / 19%	51 / 16%	0,28748*
**Spindle cell**	177 / 44%			39 / 49%	138 / 43%	
**Mixed cell**	158 / 39%			25 / 32%	133 / 41%	
**TNM (8th ed) ( No. of patients / % of patients)**						
**T1**	96 / 15%			19 / 15%	77 / 15%	0,96851*
**T2**	222 / 34%			43 / 34%	179 / 34%	
**T3**	226 / 35%			46 / 37%	180 /34%	
**T4**	100 / 16%			18 / 14%	82 / 16%	
**Stage (8th ed) ( No. of patients / % of patients)**						
**I**	76 / 11,8%			14 / 11%	62 / 12%	0,69927*
**IIA**	198 / 30,7%			44 / 35%	154 / 29%	
**IIB**	185 / 28,7%			37 / 29%	148 / 28%	
**IIIA**	143 / 22,2%			26 / 21%	117 / 22%	
**IIIB**	39 / 6,1%			5 / 4%	34 / 6%	
**IIIC**	3 / 0,5%			-	3 / 1%	
**Hypertension ( No. of patients / % of patients)**						
**Yes**		251 / 39%	49 / 39%		202 / 39%	0,87168*
**No**		334 / 52%	67 / 53%		267 / 51,5%	
**Unknown status**		59 / 9%	10 / 8%		49 / 9,5%	
**Diabetes Mellitus ( No. of patients / % of patients)**						
**Yes**		82 / 13%	14 / 11%		68 / 13%	0,69107*
**No**		490 / 76%	100 / 79%		390 / 75%	
**Unknown status**		72 / 11%	12 / 10%		60 / 12%	

*-Pearson’s chi-squared test, **Student’s t test

Taking into account the time of diagnosis of the additional primary malignancy (before or after UM), we found out that 73 patients (58% / 11% of whole group) have developed the other carcinoma post the diagnosis of UM, while 48 (38% / 7% of whole group) already presented with a history of other location of primary cancer; for 5 patients (4%) we couldn’t obtain the certain date of neoplasm diagnosis and they were excluded from further analysis. In this subset the age at primary treatment (p = 0,0454) and histological type of melanoma (p = 0,00697) proved to be associated with the occurrence of additional primary malignancy. Patients in whom additional malignancy was diagnosed after UM treatment were younger and more commonly diagnosed with spindle-cell melanomas. The demographic details of both subsets are listed in Table 4, while the detailed locations of additional carcinomas with regards to the time of diagnosis of UM are presented in Table 5. Of note, the type of UM treatment (surgery vs. radiotherapy) also had no effect on the occurrence of additional malignancy, the specific data is listed in Table 6. In our study group there was only 1 case of soft tissue sarcoma that developed in the head and neck region post the diagnosis of uveal melanoma – in patient that was primarily treated with enucleation of the globe.

**Table 4:** Subgroups analysis – depending on time of diagnosis of additional malignancies - before or after uveal melanoma (UM)

	Additional malignancies			***p-value***
	Total	before UM	after UM	
	N = 121	N = 48	N = 73	
**Gender**				
**Women**	**54 (45%)**	24 / 50%	30 / 41%	0,3351*
**Men**	**67 (55%)**	24 / 50%	43 / 59%	
**Age (years)**				
**Mean (± SD)**	61	68,98 ± 9,12	64,22 ± 11,02	0,0454**
**Tumours largest basal diameter (mm)**				
**Mean (± SD)**	12,25	12,63 ± 3,21	12,01 ± 2,41	0,3962**
**Tumours height (mm)**				
**Mean (± SD)**	7,68	7,65 ± 2,96	7,7 ± 2,14	0,9647**
**Histological type**				
**Epithelial cell**	14 /18%	5 / 17%	9 / 20%	0,00697*
**Spindle cell**	37 / 49%	9 / 30%	28 / 60%	
**Mixed cell**	25 / 33%	16 / 53%	9 / 20%	
**TNM (8th ed) ( No. of patients / % of patients)**				
**T1**	18 / 15%	9 / 18,75%	9 / 12,3%	0,09038*
**T2**	41 / 34%	10 / 20,8%	31 / 42,5%	
**T3**	45 / 37%	20 / 41,7%	25 / 34,2%	
**T4**	17 / 14%	9 / 18,75%	8 / 11%	
**Stage (8th ed) ( No. of patients / % of patients)**				
**I**	13 / 11%	5 / 10,4%	8 / 10,9%	0,296*
**IIA**	42 / 35%	12 / 25%	30 / 41,1%	
**IIB**	36 / 30%	16 / 33,3%	20 / 27,3%	
**IIIA**	25 / 21%	11 / 22,9%	14 / 19,2%	
**IIIB**	5 / 4%	4 / 8,33%	1 / 1,3%	
**IIIC**	-	0	0	

*-Pearson’s chi-squared test, **Student’s t test

**Table 5:** Location of additional malignancies in uveal melanoma (UM) patients – depending on time of diagnosis – before or after UM

ICD-10 code	Location	Total	before UM		after UM	
		n	n	%	n	%
C00	**Lip**	2	2	100%	0	0%
C16	**Stomach**	4	0	0%	4	100%
C18	**Colon**	10	0	0%	10	100%
C20	**Rectum**	3	1	33%	2	67%
C25	**Pancreas**	1	0	0%	1	100%
C32	**Larynx**	3	2	67%	1	33%
C34	**Bronchus and lung**	15	4	27%	11	73%
C43	**Malignant melanoma of skin**	2	2	100%	0	0%
C44	**Other skin malignancies**	17	**8**	**47%**	**9**	**53%**
C45	**Mesothelioma**	1	0	0%	1	100%
C49	**Malignant neoplasm of other ** **connective and soft tissue**	1	0	0%	1	100%
C50	**Breast**	17	**9**	**53%**	**8**	**47%**
C51	**Vulva**	1	0	0%	1	100%
C53	**Cervix uteri**	1	1	100%	0	0%
C54	**Corpus uteri**	6	5	83%	1	17%
C56	**Ovary**	3	0	0%	3	100%
C61	**Prostate**	13	**7**	**54%**	**6**	**46%**
C64	**Kidney**	3	3	100%	0	0%
C67	**Bladder**	10	4	40%	6	60%
C73	**Thyroid gland**	2	0	0%	2	100%
C83	**Non-follicular lymphoma**	1	0	0%	1	100%
C85	**Other specified and unspecified types of non-Hodgkin lymphoma**	1	0	0%	1	100%
C91	**Lymphoid leukemia**	1	0	0%	1	100%
C92	**Myeloid leukemia**	1	0	0%	1	100%
D03	**Melanoma in situ**	1	0	0%	1	100%
D09	**Carcinoma in situ of bladder**	1	0	0%	1	100%
		121	**48**		**73**	
			40%		60%	

**Table 6:** The treatment methods with regards to the frequency of additional malignancies in studied cohort

		Total	Additional malignancies		Others	***p-value***
			after UM	before UM		
		N = 644	N = 73	N = 48	N = 523	
**Surgical treatment**	**Enucleation**	339 / 52,6%	39 / 53%	26 / 54%	274 / 52,4%	0,92144*
	**Resection**	40 / 6,2%	5 / 6,8%	1 / 2,1%	34 / 6,5%	
	**Endoresection**	4 / 0,6%	0	0	4 / 0,8%	
	**Exenteration**	3 / 0,5%	1 / 1,4%	0	2 / 0,4%	
	**No treatment**	7 / 1,1%	0	1 / 2,1%	6 / 1,1%	
	**Total**	**393 / 61,0%**	**45 / 61,6%**	**28 / 58,3%**	**320 / 61,2%**	
**Radiation treatment**	**Ru-106 brachytherapy**	209 / 32,5%	27 / 37,0%	15 / 31,3%	167 / 31,9%	
	**TTT**	25 / 3,9%	1 / 1,4%	3 / 6,3%	21 / 4,0%	
	**SRT**	17 /2,6%	0	2 / 4,2%	15 / 2,9%	
	**Total**	**251 / 39,0%**	**28 / 38,4%**	**20 / 41,7%**	**203 / 38,8%**	

*-Pearson’s chi-squared test

## Discussion

This is a single centre observation on the frequency of additional primary malignancies among UM patients who received their primary treatment throughout the period of 26 years. We found out that 20% of patients had another malignancy prior or after the diagnosis of UM. All patients had their follow-up visits in our Department and most of them were treated in Greater Poland Cancer Centre, if they happened to develop the additional malignancy. Survival data were collected from the Greater Poland Cancer Registry in conjunction with National Cancer Registry if necessary, which proves coherent database.

There are various hypotheses dealing with occurrence of additional primary malignancies in UM patients. Because of common co-existence with cutaneous melanoma and breast cancer many reports direct attention to BAP-1 tumour predisposition syndrome (*BAP1*-TPDS) [6, 7, 10, 13]. This inherited disorder is associated with an increased risk for numerous tumours, both malignant and benign, e.g.: uveal melanoma, malignant mesothelioma, cutaneous melanoma, renal cell carcinoma and basal cell carcinoma. Other alleged tumours in *BAP1*-TPDS comprise: breast cancer, neuroendocrine carcinoma, non-small-cell lung adenocarcinoma, thyroid cancer and urinary bladder cancer [21, 22]. In affected patients more than one type of primary cancer may occur, with younger ager of onset than in the general population. Of note, in UM patients, the presence of somatic BAP-1 mutations in tumour cells increases the risk of metastasis and worsens prognosis [6, 14]. However, the frequency of germline BAP1 mutations according to many groups is low, about 2%, and cannot be related to higher frequency of additional primary malignancies [23, 24].

Recently, there have been reports of a new germline mutation associated with uveal melanoma, which may also be responsible for the development of additional malignancies, namely the mutation of the MBD4 gene (responsible for repairing DNA damage). This mutation is nowadays systematically explored, and therefore the spectrum of tumours associated with it will most likely expand, but as for now it was found in such malignancies as a polyposis-associated colorectal adenocarcinoma, a spiradenocarcinoma, a glioblastoma, a pilocytic astrocytoma, a gastric adenocarcinoma, a pancreatic adenocarcinoma and a pancreatic endocrine tumour [6, 7, 14, 17, 25, 26]. Identification of MBD4 mutation in UM patients may be important prognostic factor, as they seem to respond to immunotherapy [25, 26–28]. But it’s still not sure what is the exact frequency of this mutation among UM patients.

The higher frequency of additional malignancies in UM patients has already been reported, but the final conclusions differ depending on the source. According to some authors, the incidence is no different than in the healthy population, with rates lower than 10% [20, 22, 30, 31]. On the contrary, there are other reports suggesting that the frequency of additional malignancies is higher for the UM patients with an approximately 11% higher risk than in healthy subjects [7, 12, 13, 15, 34], ranging to even more than 20% in Scandinavian population [17, 24]. In our cohort the additional malignancy was diagnosed in 20% of registered UM patients. Most common location of other cancer was skin (15%, without distinction to histopathological types of neoplasm in Cancer Registry), followed by breast (13%) and lungs (12%). If cases of basal cell carcinoma and skin lesions were excluded from the analysis – the ratio of additional malignancies would of course be lower in our cohort, but still higher compared to most other previously published reports. According to National Cancer Registry Report in Poland, in 2019 the most common sites of cancers in men were prostate (20,6%), followed by lungs (16,1%) and colon (6,8%), and in women breast (22,9%), followed by lungs (9,9%) and corpus uteri (7,0%) [35]. In our cohort the most common locations of additional malignancies in men were prostate (20%), skin (16%) and both lungs and bladder (14%); and in women breast (29%), skin (15%) and corpus uteri (11%) (Table 2).

In further analysis, while the group was split into two, regarding the time point of diagnosis of additional malignancy, we found out that the incidence of some tumours (breast, skin, prostate) was equal in both groups (with other cancer diagnosed before or after UM), similarly to their occurrence in healthy population, whereas for other types of tumours the incidence varied significantly between groups. We observed that tumours of lips, larynx, uterus and kidney were more likely to occur before treatment of uveal melanoma; while tumours of gastrointestinal system, bladder, thyroid gland, blood system and mesothelioma were diagnosed more commonly after the treatment of uveal melanoma. The significance of this finding is of course limited due to the small number of the studied group.

Patients in whom additional malignancy was diagnosed after UM treatment were younger and more commonly diagnosed with spindle-cell melanoma. It may be speculated that this is because this type of UM seems to have better prognosis, therefore with longer survival there might be a higher possibility of developing other cancers, just as in a healthy population. Interestingly, other recently published report in this subject confirms the same observation [24].

This single centre cohort analysis reports the high frequency of additional primary malignancies in UM patients. One of the possible explanations would be some genetic predisposition to the development of other carcinomas, perhaps of specific location, however due to the retrospective character of the study, the chromosomal analyses were not performed.

## Conclusions

The incidence of additional malignancies was higher in our cohort of UM patients than reported by most of other groups. Patients who developed additional neoplasm post the diagnosis of UM were younger and had more spindle cell tumours than those who presented with already diagnosed neoplasm. If there is a certain predisposition to a specific type of additional primary malignancy in UM patients, the analysis of larger database is required.

## Data Availability

The datasets used and/or analysed during the current study available from the corresponding author on reasonable request.
